# Stem cell culture conditions and stability: a joint workshop of the PluriMes Consortium and Pluripotent Stem Cell Platform

**DOI:** 10.2217/rme-2019-0001

**Published:** 2019-04-02

**Authors:** GN Stacey, PW Andrews, I Barbaric, C Boiers, A Chandra, G Cossu, L Csontos, TJR Frith, JA Halliwell, ZA Hewitt, M McCall, HD Moore, M Parmar, MB Panico, V Pisupati, V Shichkin, AR Stacey, FS Tedesco, O Thompson, R Wagey

**Keywords:** Cell therapy, pluripotent stem cells, mesenchymal cells, clinical trial, characterization regulation, manufacturing, regenerative medicine, cell-based medicines

## Abstract

Human stem cells have the potential to transform medicine. However, hurdles remain to ensure that manufacturing processes produce safe and effective products. A thorough understanding of the biological processes occurring during manufacture is fundamental to assuring these qualities and thus, their acceptability to regulators and clinicians. Leaders in both human pluripotent and somatic stem cells, were brought together with experts in clinical translation, bio-manufacturing and regulation, to discuss key issues in assuring appropriate manufacturing conditions for delivery of effective and safe products from these cell types. This report summarizes the key issues discussed and records consensus reached by delegates and emphasizes the need for accurate language and nomenclature in the scientific discourse around stem cells.

**Lay abstract:**

Novel therapies derived from different kinds of precursor cells and stem cells are increasingly moving to clinical trials to restore tissue function in patients who have suffered injury or disease. The manufacture of these new therapies is unusually complex, which means that the manufacturing processes require great attention to assure they are safe and effective. This paper describes a conversation amongst experts in the field who are exploring therapeutic applications of two different kinds of stem cells (pluripotent stem cells and tissue-derived stem/precursor cells). It considers critical issues in developing the manufacturing process for each of these quite different cells types.

## Introduction

A workshop on the culture and stability of human pluripotent stem cells (hPSCs) and what have been called “mesenchymal stromal cells” (MSCs) for clinical application was co-organised by the UK Regenerative Medicine Platform Pluripotent Stem Cell Platform [1] and the EU-funded PluriMes project [2] at Madingley Hall, University of Cambridge (UK; 28-29^th^ November 2016). The meeting participants included basic researchers, culture reagent manufacturers, clinical trial leads and the UK regulatory body, the Medicines and Healthcare products Regulatory Agency (MHRA). The meeting addressed a range of key issues in the *in vitro* culture and stability of hPSCs and tissue specific mesenchymal cells with multipotent capacity for clinical use. Workshop sessions debated fundamental issues in stem cell nomenclature and metrics, raw materials and stem cell manufacturing processes. Here we summarise the key issues raised by and findings of, each speaker and the conclusions and consensus drawn from each discussion session.

## Session 1: Maintaining stem cell state *in vitro*: the impact of medium and matrix on undifferentiated state

### From derivation to robust defined culture of human embryonic stem cells for therapeutic applications

Harry Moore (University of Sheffield, UK) described the process of developing human embryonic stem cells (hESCs) for clinical use under EU and UK regulation. In particular, he addressed the requirement for detailed record keeping and risk assessment of all materials that could affect the quality and safety of such cell lines. Moore also described the challenges of, and progress towards, better standardised culture conditions for hESC lines including the use of fully defined growth media and extracellular matrices and the importance of careful validation of new methods and their impact on the final product. Also described were some interesting effects of culture of hESCs on synthetic gels (Polyglycerol monomethacrylate 2-hydroxypropyl methacrylate (PGMA-PHPMA) gels), also called “worm” gels. These appeared to mimic natural mucins and induce a state in hESC cultures similar to diapause seen in embryos representing a “resting” state which may have potential in engineering more stable pluripotent hESC cultures [3].

Moore emphasised the crucial importance of work performed on hESCs as a gold standard for the development of regenerative medicines that will follow based on induced pluripotent stem cell (iPSC) lines. hPSCs prepared with the intention of use in therapy would have to meet the requirements of the European Union Tissues and Cells Directive [4, 5]. He also described the process of “rederivation” denoting a cell line derived for research purposes which had been prepared under controlled clean room conditions as candidate cell stock for use in development of regenerative medicines. However, this process alone was not considered sufficient to establish a cell line as suitable for use in clinical trials and all lines intended for this purpose must be shown to meet the requirements of the EU Tissue and Cell Directive (EUTCD). In regulating clinical trials and development of product Market Authorisations, the MHRA expected regulatory compliance with EU requirements for such starting materials; i.e., EUTCD. Group discussion also reflected on the fact that donors may withdraw consent and in some cases in the EU this had meant that hESC lines developed for clinical use had been withdrawn. However, this was not a pan-EU requirement and in the UK, withdrawal of donor consent for use of their tissue would only affect the original donated tissue and not its derivatives such as hPSC lines.

### Mutation rate, epigenetics, and selective advantage in human pluripotent stem cell cultures

Oliver Thompson (University of Sheffield, UK) explained that the drivers for mutation in hPSCs were poorly understood and difficult to predict, but the risk of such events could be reduced through the use of good cell culture practice. Thompson described whole genome sequencing, DNA methylation (bisulphite sequencing) and RNAseq data gathered from 80 clonal hESC cultures (from two clinical grade hESC lines, MShef4 and MShef11) maintained for 3 months, comparing standard normoxic conditions with and without Rho kinase (ROCK) inhibitor and hypoxic conditions. Somatic mutations had decreased under low oxygen conditions (i.e., 5% oxygen) whereas ROCK inhibitor and both nominally stable and unstable cell lines had shown a similar number of mutations. Additionally, a number of differentially expressed genes were identified by RNAseq analysis. This had revealed that their expression differed most significantly between different cell lines and less so with changes in culture conditions. In particular, subtle differences in the expression of lineage-specific genes was observed, which required follow-up testing to look for bias in lineage differentiation capacity. Ongoing work sought to resolve relationships between DNA methylation and gene expression with the incidence of genomic changes. Thompson concluded that key elements of handling hPSCs to ensure reproducible and stable features were the use of early passage cells, careful and gentle passage to avoid cell damage, frequent changes of growth media and minimising the number of cryopreservation steps for the cells used in experimental work.

### Detecting genetic changes in cultures of human pluripotent stem cells

Ivana Barbaric (University of Sheffield, UK) focused on the issue of genetic stability of hESC lines, describing the most common karyotypic changes as gains of chromosomal material. Indeed, over 70% of all reported karyotypic changes are gains of either whole chromosomes or chromosomal regions [6], and the most frequently affected chromosomes in hESCs include chromosomes 1, 12, 17, 20 and X. Amongst the effects of such changes on stem cell behaviour are competitive growth advantage enabling variants to outcompete the wild type cells and take over the culture, altered or reduced potential for differentiation and potential malignant transformation [7, 8, 9]. In the light of such findings, Barbaric highlighted the need to detect rare variants when they appear in cultures. To that end, she contrasted the range of techniques available for the analysis of genome integrity in hESC lines. Despite the breadth of methodologies available, it is apparent that no single technology is ideal, as the issues of sensitivity, resolution, high-throughput and cost hamper different methodologies to varying extents. Commonly used techniques such as karyotyping, fluorescence in situ hybridization (FISH) and quantitative PCR (qPCR), generally fail to detect variant cells when they are present at low frequencies in hESC cultures (less than 5-10%) [6]. Hence, the challenge remains to increase levels of detection for clinical cell-based products. Barbaric also emphasised that in addition to the ability to detect genetic changes with high precision and sensitivity, it is pivotal to identify culture conditions that would minimise genetic changes from appearing and expanding in hESC cultures, hence enabling large scale production of genetically stable hESC for applications in regenerative medicine and drug discovery. Nonetheless, we ultimately need the knowledge of which particular variants are potentially detrimental and which are non-consequential for uses in regenerative medicine. Thus, deciphering the functional consequences of genetic changes will be an important topic to be resolved for the safety assessment of regenerative medicine therapies.

### Defined cell culture workflow to enhance consistency for derivation and maintenance of (“stable”) mesenchymal progenitors from human pluripotent stem cells

Ravenska Wagey (STEMCELL Technologies, Canada) described a workflow for the derivation, expansion and cryopreservation of mesenchymal progenitor-like cells (MPCs) from human pluripotent stem cells (hPSCs) in a defined culture system. The differentiating cells lost their hPSC markers and adopted MPC morphology by day 7. These cells formed colony forming unit-fibroblasts (CFU-F) and by day 21 stably expressed the “typical” MPC surface markers. The hPSC-derived MPCs in this defined culture medium displayed robust cell expansion with a doubling time of 1.1-1.4 days for over 17 passages (P) and were capable of differentiating *in vitro* to osteoblast (Alizarin red positive), adipocyte (Oil red O positive) and chondrocyte (Alcian blue positive) lineages as a surrogate for *in vivo* transplantation. At any stage of the cell expansion phase, these hPSC-derived MPCs can be cryopreserved in a defined cryopreservation medium (MesenCult ™-ACF Freezing medium or CryoStor CS10) with high cell recovery and viability post-thaw. These cryopreserved cells maintained “normal” karyotype (100% diploid) at early and late passages. However, it was noted that passage number and other stress factors in culture could have an effect on maintaining a “stable” karyotype and the *in vitro* differentiation capacity of these cells. Wagey emphasised that it was important not to underestimate the “culture system” as it could impact cell quality and potency. Currently, these hPCS-derived MPCs are being evaluated for their potential to form bone and chondrocytes in animal models which, are considered to be the current gold standard assays for differentiation potential of these cells. Analysis by qPCR is conducted to provide insight on the transcriptome profile of these cells.

Key quality criteria used to assess hPSC-derived MPCs in cultures are detailed in Box 1.

Wagey considered that evaluation of cell function in animal models is a crucial step towards validating the *in vitro* data to demonstrate therapeutic potential and this work is now underway at STEMCELL Technologies in collaboration with Mara Riminucci (Sapienza University of Rome, Italy).

### Human pluripotent stem cell-derived myogenic cells: "reprogramming" experimental therapies for muscle disorders

Francesco Saverio Tedesco (University College London, UK) described the challenge of treating muscular dystrophies. This group of disorders affect skeletal muscle (the most abundant human tissue) and is characterised by muscle wasting, exhaustion of endogenous skeletal muscle stem cells and lacks effective treatments. New approaches to provide therapies included mutation-specific strategies (e.g., antisense-oligonucleotide-mediated exon-skipping) and genetic interventions (e.g., gene replacement and gene editing). Genetic interventions suffer from the challenge of the large size of some genes and the numerous mutations which may be involved, such as in Duchene muscular dystrophy. Tedesco reviewed the possibility of cell therapies based on use of a range of cell types from different tissues, focusing on muscle-derived myoblasts, mesoangioblasts and CD133+ cells. Key challenges identified for therapies based on these cells were lack of availability of donor tissue, exhaustion of endogenous stem cells, proliferation capacity and clinical delivery of therapeutic cells. He described an important potential solution to many of these issues by generating similar cell types using cultures differentiated from human iPSC lines. Tedesco described the successful use of gene-corrected iPSC-derived inducible myogenic cells in a mouse model for Limb-Girdle Muscular Dystrophy [10] and identified the key characteristics required for such cells as: 1) they must be stably expandable, 2) capable of efficient differentiation and fusion with host myofibers, 3) amenable to genetic correction, 4) systemically deliverable in the patient, 5) non-tumorigenic and 6) able to persist and ideally, self-renew *in vivo.* Finally, Tedesco described ongoing work to develop transgene-free iPSC-derived myogenic cells and validate differentiation protocols in order to enable development of cells and protocols suitable for clinical use.

### Discussion session 1: workshop discussion on stem cell state

#### *Can the “pluripotent” or “multi-potent” states be suitably characterised by biological and/or* physical measurement?

Delegates agreed that a functional definition for pluripotent cells was “those cells with the capability to differentiate into all somatic cell types”. However, participants also concluded that options for functional assessment of hPSCs are limited in the absence of a human equivalent of the mouse embryo complementation assay. Teratoma formation in immunocompromised mice, formation of embryoid bodies and *in vitro* differentiation were commonly used to demonstrate the capacity of hPSC lines to generate exemplar cells representing each germ layer. In addition, the Pluritest™ assay applied to undifferentiated cultures was used quite widely and attempted to predict the property of pluripotency without an *in vitro* differentiation step.

Delegates concluded that the term “pluripotency markers” was misleading for cell markers such as TRA-1-60 and Oct-4. Such markers are also expressed by other cell types, thus, expression of specific markers whilst possibly necessary for a pluripotent state will not confirm whether an individual cell or population is pluripotent or not. Furthermore, mutations in the genes associated with some markers expressed by stem cells, do not appear to have an adverse phenotype. Whilst current pluripotency assays based on *in vitro* differentiation in some cases may give equivocal or inconsistent results [11], it was concluded that assays that demonstrate functional differentiation are still needed to assess pluripotent potential and that surface markers found on pluripotent cells could be described as “pluripotency-associated” markers.

The question was also asked: “Do hPSCs in the naïve state have an advantage over “primed” hPSCs for therapeutic applications?” The difficulties in distinguishing the “primed” and “naive” states and the absence of an unequivocal assay for pluripotency, make this challenging to resolve. It was concluded that in principle, cells in the naïve state could offer more reliable and effective sources for a wider range of therapeutic cell types than primed hPSCs.

#### Defining multipotency in non-pluripotent cells

For fibroblast cells often grouped together as mesenchymal progenitor cells (sometimes referred to as mesenchymal stromal cells), there was general agreement that the issues of tissue of origin, donor variability, the lack of specific markers and standardised growth media, the lack of clonal and biological studies to help understand the heterogeneity and potency of these cell populations, continue to limit progress in manufacturing cell therapies with these cells. In addition, progress in the field is made more difficult due to widespread misreporting of the so-called single or universal entity called a “mesenchymal stem cell” [12]: where in some cases these reports lack references to the biology of the cells to better define their multipotency in more rigorous assays. Standards proposed for characterisation of bone marrow derived MSCs (e.g. [13]) may not be fully applicable to MSCs derived from other tissues. Furthermore, it is not clear, based on rigorous assays, that many cells called MSCs are in fact stem/progenitor cells. Better functional assays are needed to assess the impact of any changes to these cell populations during *in vitro* culture. It should be noted that recently, a more accurate nomenclature for mesenchymal progenitor or mesenchymal stromal cells has been proposed [14], which addresses the issues discussed above and puts forward the term of “tissue specific stem/progenitor cells” to describe these cell types derived from different tissues.

#### Conditions for stable cultures

Establishing defined and stable culture conditions is fundamental to achieving the reproducibility and consistency required to manufacture cell therapies under a good manufacturing practice (GMP) license. The delegates agreed that genetic changes are inherent in cell cultures and there was no evidence that pluripotent stem cells and MSCs are substantially different from other cells in the general perspective of genetic stability. However, the potential impact of genetic change on safety and efficacy of stem cell derived therapies is not clear and requires further coordination across the field to identify suitable and sensitive analytical tools, collate suitable data on genetic stability and interpret the changes observed in terms of patient safety [15]. It was clear that translating a culture system to new kinds of growth media and/or surface matrix can lead to changes in the cell culture and the use of the term “adaptation” in these circumstances may be misleading as such situations may in fact select cell populations that are irreversibly different to the original cultures.

It was clear that the number of clinical trials with MSCs had risen dramatically in recent years and the number of trials based on hPSC is also increasing. A major challenge for these novel stem cell therapies is that there is very little time between new research discoveries and attempts to implement their benefits in regenerative medicines. It should also be pointed out that many of these clinical trials do not appear to be linked to stemness, but rather to the ability of the cells secrete paracrine, immunomodulatory and immunosuppressive substances.

Establishing some understanding of the mode of action for each new stem cell-based product will be important, and in all cases, it will be important for the manufacturer to implement relevant functional assays to develop a better understanding of the cell biology in the manufacturing process and what affects it.

## Session 2: Developing reagents and cells for clinical application

### Phase 1 trials: a regulatory perspective

Maria Beatrice Panico of the Medicines and Healthcare Regulatory Authority (MHRA; UK), described the process of making an application for a Clinical Trial Authorization (CTA) this required a number of supporting documents that should consider the type of product, the phase of development, the clinical condition to be treated and route of delivery. In phase 1 clinical trials, Panico also explained the multidisciplinary approach taken by the MHRA and the need to submit a separate proposal to a Research Ethics Committee as well as consulting with MHRA Clinical Trials Unit on First Time In Human (FTIH) and First Time IN UK trials and the European guidelines on FTIH. Raw materials and starting materials such as integrating rDNA and the stem cell line required careful due diligence regarding their history of development. Panico also emphasised the extensive non-clinical testing needed to demonstrate that a new product is considered safe for use and that the manufacturer has considered genetic stability and immunogenicity. Also important was the need to ensure that pre-clinical material and manufacturing processes are consistent with those used for the clinical trial. It is vital that each manufacturer understands the biology of their product and this means that characterisation should be consistent with appropriate and current research methods and knowledge. Panico also described the process by which CTA applications are assessed by multidisciplinary teams of the regulatory body both nationally and at the European Medicines Agency (EMA). Furthermore, for high risk trials, advice from the Expert Advisory Group (EAG)/Commission on Human Medicines (CHM) may also be sought [16]. Panico went on to emphasise that product developers should seek regulatory advice from the earliest stage and maintain these channels of communication at key points throughout the product development and to be open about areas of uncertainty or concern. This iterative process would help identify any issues and enable any necessary solutions to be developed before they become critical.

### Experiences in developing reagents for cell therapy: translation from the researcher to the media manufacturer and acceptability criteria for clinical trials from researchers

Lynn Csontos (STEMCELL Technologies, Vancouver, Canada) described key elements in navigating the regulatory landscape of ancillary materials and raw materials for use in cellular therapies. These materials include cell culture media, cell separation reagents, containers, filters, etc., which are not intended to be part of the final cellular product. She explained that there is currently no global standard for raw materials and each manufacturer is responsible for the selection and use of individual reagents and components used in the manufacturing process. It was also noted however, that suppliers also have a responsibility to provide a reagent that meets any agreed specification consistently and give relevant information that will facilitate a robust risk assessment of their product. Suppliers may sometimes seek to use quality terms such as “GMP-grade” which may not meet full requirements of a regulator on review of a clinical trial application or market authorisation. Therefore, product developers should ensure due diligence to qualify all raw materials following a process of risk assessment and determination of the veracity of any claims made by suppliers regarding their quality. This will include relevant jurisdictional standards, vendor regulatory compliance and indirect involvement of animal-derived reagents in comparison with the clinical application (i.e., topical or injected). Csontos described how GMP is intended to assure the consistency of product identity, potency, purity and overall quality of a product and thereby help to assure the safety of a therapeutic. STEMCELL Technologies has therefore committed to developing high quality media and are working towards launching GMP compliant media (i.e., MesenCult™-ACF and STEMdiff-ACF™) and is currently supporting more than 30 clinical trials around the world.

### Towards a stem cell-based therapy for Parkinson’s Disease (PD)

Clinical trials using cells derived from human fetal ventral mesencephalon (VM) have shown that transplanted dopaminergic (DA) neurons can functionally reinnervate the denervated striatum, restore DA release, and in some PD patients, provide substantial long-term clinical benefit. However, the use of fetal tissue presents ethical issues and is not adequately scaleable to enable widespread use of fetal tissue transplantation for PD treatment. Malin Parmar (Wallenberg Neuroscience Center, Sweden) described the recent achievements at the University of Lund, Sweden, in manufacturing functional dopamine neurons from hESCs. The Lund group had verified that hESC-derived DA neurons were equivalent to fetal DA neurons: they can project sufficiently distant axons for use in humans, regenerate midbrain-to-forebrain projections, and innervate correct target structures. However, before these cells can be used as a cell therapy for Parkinson’s Disease they need to be produced under GMP conditions and detailed functional properties of GMP produced cells must be tested. Parmar illustrated how GMP hESC-derived midbrain DA neurons in a rat model of PD had shown long-term survival and functionality, also demonstrated efficacy in restoration of motor function with a potency comparable to that seen with human fetal DA neurons. This work had also been developed to provide predictive markers of graft success using RNA sequencing on cells transplanted at different centres [17]. Parmar emphasised the critical and time-consuming process of careful development and proofing of GMP protocols and potency assays. A fully GMP compliant differentiation protocol has been established in Lund that results in high yield differentiation of functional DA neurons after transplantation for multiple GMP hESC lines. However, the translational phase, from validation of all research protocols to GMP process, to finally be ready for making a GMP batch had taken more than 5 years. Parmar is now working towards a clinical trial scheduled for 2020 with cell manufacturing beginning in 2018 using the Roslin Cells RC17 cell line made under the requirements of the European Union Tissues and Cells Directive under the requirements of GMP.

### Pluripotent Stem Cell Platform perspectives on safety and raw materials

The presentation by Glyn Stacey (International Stem Cell Banking Initiative, UK) was based on an earlier Pluripotent Stem Cell Platform (PSCP; UK) workshop on the scientific assessment of safety for cell therapy products [18], which engaged clinical trial developers, cell therapy companies, regulators and funding bodies. It aimed to develop a clearer understanding of the potential hazards that arise for cell therapies and what new methodologies might be needed to assess and control these risks. The biological nature of cellular therapies makes them subject to variation, prone to microbial contamination and are also capable of unpredictable responses to their environment. This can impact on the consistency of stem cell differentiation, phenotypic stability, genetic stability and tumorigenicity of stem cell-derived products. This situation requires careful selection of appropriate biomarkers that relate to function and safety. The challenge of eliminating undifferentiated cells in the final product was also identified as a key challenge for product developers as was the need to establish long term follow up for patients. The workshop had also considered the use of cell tracking methods such as nanoparticles to study biodistribution in preclinical animal models. This work was progressing to illustrate not only cell location but also cell state/function, using non-toxic nanoparticles markers which in the future might be used for *in vivo* clinical tracking.

### Autologous thymic tissue as potential source for stem cell therapy

Valentin Shichkin (University “Ukraine”) described work performed in the Thymistem project [19], to generate procedures for the collection, processing and storage of thymic epithelial tissues, which could be used to reconstitute T-cell immune system functions in thymectomized patients. 80 neonates and young children under 3 years undergoing thymectomy as part of cardiac surgery, provided thymic tissue for experimental work to select an optimal tissue processing methodology comparing 8 different cryoprotective reagents containing different combinations of dimethylsulphoxide (DMSO), glycerol, sucrose, hydroxyethyl starch (HES), Dextran 40 and a commercial serum-free medium (Stem-CellBanker (AMS Biotechnology, UK). In addition, three culture media were compared: an RPMI-1640-based medium containing fetal calf serum (5 or 10% FCS) and a serum-free DMEM/F12-based medium. In serum-free culture, thymic epithelial cells showed slower growth rates and greater loss of cell viability than in serum-containing media. However, Shichkin proposed that serum-free conditions may still be suitable for short term culture of thymic epithelial cells. The study had also compared different tissue preparation methods from small (1-2 mm) and large (0.8-1.0cm) tissue fragments to cell suspensions (prepared either using mechanical or enzymatic disaggregation) and long-term stromal cell cultures. Two preservation media containing DMSO (5%)/HES(6%) (CPM-6) or DMSO (5%)/Dextran 40 (5%) (CPM-7) gave best recoveries for both disaggregation methods of which enzymic method yielded higher viable cell numbers. Satisfactory recovery of CD326 positive thymic epithelial cells was optimal in CPM-7 preserved cells. Shichkin concluded that the CPM-7 preservation medium was the most suitable for long-term storage of both thymic suspensions and thymic fragments for clinical use, according to assessment of thymic epithelia cell populations (CD326+), viability by multiple assays and stromal epithelial cell monolayer formation. Shichkin’s observations on cell preparations stored for up to two years indicated that the proportion of viable cells on thawing should be at least 20 - 25% to assure reliable recovery of stromal-epithelial cell monolayers. His work emphasised the importance of careful development of preservation conditions for such cell types which could form the basis for reliable cell therapies [20].

### Discussion session 2: Workshop discussion on raw- and starting-materials

Discussion focused on risk assessment of raw materials, where key factors were seen to be microbiological contamination and variability of biological materials. It was recognised that risk associated with individual materials may be considered differently depending on the type of therapy intended (e.g., patient cohort, repeat dose therapy) and aspects of the manufacturing process that increased exposure to materials e.g., culture passages required, volume/concentration of reagents used. Risks associated with the starting materials (cells and vectors) focused on infection risk and tumorigenicity. It was agreed that important factors in assessing risk included cells/dose and age of patients. It was pointed out that these and other issues for safety of pluripotent stem cell-based therapies had been reviewed in earlier PSCP workshops [18, 21] and special issues relating to the use of pluripotent stem cells had been addressed by the International Stem Cell Banking Initiative [22]. It was concluded that all reagents should be included in a risk assessment matrix and evaluated, but that more detailed investigations may be focussed where risk was deemed to be higher. For biological materials, the potential for batch to batch variation and microbial contamination required careful establishment of raw material specifications and in some cases even pre-use testing. Furthermore, it was felt important not to overlook those materials of non-mammalian origin, which could still harbour organisms infectious for humans. Overall responsibility for assuring safety of raw materials and starting materials clearly lay with the manufacturer, but that some responsibility was shared by suppliers who should supply to the manufacturers specification and could greatly facilitate robust risk assessment. It was recognised that regulatory advice on risk assessment was available [23] and specific queries could be put to multiple UK regulators via the MHRA Innovation Office [24]. A further key take-home message from the discussion was that in the EU, GMP raw materials are expected to be used where available for clinical studies. This is subtly different from expectations in North America and could be a key issue for successful translation of raw material products from a North American to a European setting.

## Session 3: Introduction to manufacturing issues

Glyn Stacey introduced some of the key issues in the development of seed stocks of cells and the importance of identifying Critical Quality Attributes (CQAs) of the product to help set appropriate quality controls [25]. It is also important to implement a quality assurance framework that applies over the whole process from source materials to biobanking, storage and shipment. Other key aspects of manufacturing were identified as key contributors to a robust risk assessment (see above) and recognition that monitoring and testing will be required at different stages, not just at the point of product release. This was felt to be important to give confidence that the cell therapy will be consistent and bears acceptable risk regarding both patient safety and developer investment.

Stacey also described a hierarchy of standards starting with regulations and laws. These were supported by regulatory guidance [26, 27] and other documents such as European Pharmacopoeia monographs and general chapters [28], which were available to assist manufacturers in achieving compliance with the regulations. Finally, Stacey described the importance of scientific and ethical best practice and vigilance for emerging issues. If neglected these issues could put patients at risk of infection or malignancy and damage developers including loss of reputation. The development of such best practice documents was considered most valuable when it was accurately scoped, avoided duplication or overlap with existing best practice and engaged key experts from the scientific, regulatory and industrial community. Stacey also emphasised that publication of a best practice document is just the beginning of a pathway to implementation, which included endorsement by professional bodies, policy makers and funding agencies and uptake in local practice. Examples were cited of established best practice for cell culture [29, 30] and establishment of pluripotent stem cells for clinical use [22].

### Problems in manufacturing adult stem cells as advanced therapy medicinal products (ATMPs)

Giulio Cossu (University of Manchester, UK) illustrated challenges in manufacturing adult stem cell products from his perspective as a researcher having to adapt to the demands of a clinical trial. The practical example used was a phase 1/2a trial of donor mesangioblasts from human leukocyte antigen (HLA)-matched donors applied intra-arterially to treat Duchene muscular dystrophy [31]. Mesoangioblast cells are mesoderm progenitors derived from post-natal muscle tissue and have the capacity to differentiate into both smooth and skeletal muscles. The therapy in question was considered an ATMP as the cells had been subjected to substantial manipulation in processing and cell culture. It was considered relatively safe but had shown low efficacy to date.

Cossu identified three key challenges in establishing the clinical trial, which were biological, logistic and regulatory issues. Biological problems focused around the variability of cell recovery, proliferation and their differentiation efficiency. The latter meant that a magnetic bead selection process may be needed with some donor samples to remove undesired cell types. Logistic problems were primarily associated with safety due to viral contamination of fetal calf serum (FCS) requiring sourcing of irradiated serum, variability in the composition of culture medium and sudden withdrawal of supply of the growth medium by its manufacturer which required emergency changes to the protocol for two patients, requiring careful risk assessment. Whilst the manufacturer (Sigma-Aldrich) agreed to continue supply for the trial, the cost was increased substantially, due to replacement of bovine FGF with human recombinant FGF. Cossu’s group identified and evaluated a new cell culture media (MyoCult™-SF) which removed the need to use FCS and better supported proliferation of mesangioblast cells. Unfortunately, whilst this seemed a positive move to improve safety and product consistency, the introduction of a new growth medium required significant validation work causing further delays.

Latest developments involved the use of lentiviral vectors to express a snRNA that corrects the reading of the dystrophin gene in the patient’s own mesangioblasts, recovering normal dystrophin expression when returned to the patient. This is judged a new ATMP requiring revalidation. This has been approached by utilising the ability to cryopreserve the cells at an intermediate product stage to enable time for the cell processing protocol to be approved.

Finally, in order to enhance cell reproducibility, the project progressed to incorporate transduction of the mesangioblasts with a lentiviral vector expressing telomerase to immortalise the cells. This proposed change, still in a pre-clinical stage, raised a significant regulatory challenge to validate the use of the transduced cells as it is considered a new Investigational Medicinal Product (IMP) even though the treatment was primarily eliciting a pharmacological and immunological therapeutic effect. In addition, this new process also raised safety concerns that genetic transformation and very strong proliferation might indicate the development of malignant cell types. Additional validation will therefore be required to show that the product did not cause tumours in mice and could still deliver the required engraftment and therapeutic impact.

### Manufacturing operational constraints and their impact on commercially sustainable regenerative medicine development

Mark McCall (Loughborough University, UK) highlighted the challenges in translation of research stage cell therapy into clinical development, particularly scale-up in a GMP facility. The numerous and variable input materials to produce a complex cell-based product of suitable quality involve developing a unique manufacturing model with a novel production process for each new product. McCall discussed how to develop control strategies for complex response profiles. He also reflected on the crucial differences between research lab processes and scaled up manufacturing in a GMP environment, which often include reactor vessel dynamics and temperature control. Increased culture vessel scale for GMP manufacturing requires close attention to culture feeding regime that impacts on cell proliferation rate. To support this McCall explained that quantitative monitoring of bioreactor head space gas (i.e. dO_2_) and metabolites including glucose and lactate, should be part of the production process for quality monitoring. It was clear that all of these factors could affect the quality of the cell component in the end product and thus it is critical to understanding of input cell quality in relation to process yield. Appropriate quality systems are central to delivery of reproducible product and become additionally significant when dealing with multiple manufacturing sites to ensure integration of standards for cell expansion, differentiation, cryopreservation and characterization. Quantification of cell culture is vital to understand the manufacturing process but is also challenging due to the potential for variation in results between laboratories and different methods. Overall consistency in process development can be promoted by adopting common sources of cells (i.e., master and working cell banks) and key process methodologies such as passaging, preservation and thawing as well as cell counting. Finally, McCall directed delegates to guidance from the International Conference on Harmonisation (ICH) [32] regarding various aspects of risk assessment and process development.

### Discussion session 3: Workshop discussion on manufacturing issues

Minimizing cell culture manipulation and careful assessment of raw materials were considered key issues in helping to assure safety and quality of stem cell-based products. Tools such as Quality by Design (QbD) were concluded to have a potentially important role to play in improving established protocols for cell-based manufacture. The identification of relevant CQAs [21] was considered crucial in developing a final manufacturing and quality control framework. A further issue raised in relation to quality control was the use of external testing services. Delegates agreed that great care was required in selecting such contractors to ensure that test methods were validated with relevant test items and that all testing is performed under the appropriate standard, which is typically good laboratory practice (GLP) [33, 34].

It was clear from the presentations of Parmar and Cossu that development of manufacturing protocols, identification of CQAs, quality control assay development and even slight modifications to clinical trials protocols could be demanding and take considerable time. This could mean that there is a challenging ten-year development process from research protocol to GMP process. In conclusion, it was agreed that in developing cell therapies it was crucial to have identified the putative mode of action, to understand the biology involved in the manufacturing process and to have considered the whole process at outset, including the means of delivery to the patient.

The group recommended that in order to meet these challenges consensus guidance was required on cell therapy manufacturing which would be vital for academic groups involved in developing cell therapy products. Efforts such as the PSCP workshop programme and its links with organisations such as the International Alliance for Biological Standardisation were supporting the development of such guidance [35, 36].

## Future Perspective

It is clear that there is today, active product development of stem and progenitor cells from different tissues and pluripotent stem cell lines. Many clinical trials for cell therapies already exist for the use of tissue-derived stem or progenitor cells often misdescribed as “mesenchymal stem cells”. There is an urgent need to rationalise the biologically different cell types which have been described under this unhelpful and inaccurate nomenclature, in order for their clinical potential to be more efficiently realised in safe and reliable therapies. Pluripotent stem cells have given their first tentative indications of efficacy in one trial and many more are close to clinic. There are clearly challenges to identify the most relevant biomarkers of stem cells cultures and their derivatives to assure consistent manufacturing processes, function of the final products. Such tools will also be vital to enable manufacturers to assess the impact of changes in bioprocessing, including roll out to large patient groups, through multiple manufacturing sites. A clear challenge to manufacturers is the need to understand the biological nature of the cells they are using as manufacturing substrates and their stability *in vitro*. The UK Regenerative Medicine programme continues to explore these issues and hopefully, will deliver further outputs on this emerging field of advanced therapeutics.

## Box 1 Quality criteria for the assessment of hPSC-derived MPCs in culture

◦ Loss of embryonic markers (TRA-1-60, Oct-4) and adoption of mesenchymal-like cell morphology by day 7
◦ Starting cultures exhibit colony formation by CFU-Fs and robust cell expansion capacity for over 10 passages (doubling times of 1.1-1.5 days)
◦ hPSC-derived MPCs stably expressed MPC markers (CD105/CD73/CD146) long-term
◦ Differentiate *in vitro* into adipogenic, osteogenic and chondrogenic cells
◦ Post-cryopreserved cells maintain high recovery, viability and proliferation potentials.
◦ Cells exhibited diploid karyotype throughout the culture period

## Executive Summary

Key issues for the development of stem cell-based medicines:

- Careful development of culture medium, surface matrix and gas atmosphere is crucial to facilitate stable expansion of consistent stem cell cultures. During selection of suitable stem cell lines for manufacture, product developers may also need to consider the variation in stability of different cell lines.
- Optimal analytical regimes for the detection of genetically altered stem clones have yet to be resolved. Furthermore, a better understanding of the various genetic changes that may arise in stem cell cultures is required before their real significance for patient safety can be determined.
- Assays of stem cell functionality are crucial to determining maintenance of stem cell nature *in vitro* and it is important to understand the limitations of chosen assays for determining stem cell potency. Some form of cellular differentiation step was considered an important in assessment of pluripotent potential. Furthermore, it was agreed that the term “pluripotency markers” was misleading when applied to cellular markers associated with stem cell phenotype and self-renewal since these are shared with other non-pluripotent cell types.
- Tissue-derived stem/progenitor cells such as mesangioblasts and also iPSC-derived cells such as myogenic progenitor cells, have clear potential for development of therapeutic cells for unmet medical needs such as muscular dystrophy.
- It was evident that numerous cell therapies including many so-called “mesenchymal stem cell” treatments did not appear to require stem cell properties and relevant assays of product function were crucial to the reliable use of such cell types for therapy. Furthermore, it was recommended that the term “mesenchymal stem cell” should be replaced by more accurate descriptions of the specific cell types in question.
- Selection of appropriate raw materials requires careful risk assessment of sources and it was important for product developers to understand the requirements for regulatory acceptance of these and cell sources intended for use in manufacturing cell therapies. Early and iterative interaction with regulators were considered vital to help early product developers make most efficient progress to market authorisation.
- The development of a manufacturing process for stem cell culture derived products will require careful evaluation of bioanalytics to assure their relevance to therapeutic function and rarely product developers should be prepared to continue process development into clinical trial due to the complexity and rapid development in this field of regenerative medicine.
- Risk assessment and management of risk, for the entire manufacturing process, is important for the delivery of safe and effective products. In addition, establishment of appropriate Critical Quality Attributes and the understanding of potential impact of process change on biological function, was especially challenging for manufacture of cell therapies given the challenge of establishing meaningful biometric assays.
